# Effect of a multi-dimensional case management model on anti-retroviral therapy-related outcomes among people living with human immunodeficiency virus in Beijing, China

**DOI:** 10.1186/s12879-020-05219-9

**Published:** 2020-07-09

**Authors:** Lili Dai, Xiaochun Yu, Ying Shao, Yali Wang, Zaicun Li, Jiangzhu Ye, Shaoli Bai, Xiaoling Guo, Jianyun Wang, Bin Su, Taiyi Jiang, Tong Zhang, Hao Wu, Sarah Robbins Scott, An Liu, Lijun Sun

**Affiliations:** 1 Center for Infectious Diseases, Beijing Youan Hospital, Capital Medical University, Beijing, 100069 China; 2 School of Public Health, Anhui Medical University, Hefei, 230032 Anhui China; 3 Lanzhou Municipality Pulmonary Hospital, Lanzhou, 730046 Gan Su China; 4 The National Center for AIDS/STD Control and Prevention, Chinese Center for Disease Control and Prevention, Beijing, 100037 China

**Keywords:** Case management, HIV, Virologic failure, Retention rate

## Abstract

**Background:**

This paper introduces a comprehensive case management model uniting doctors, nurses, and non-governmental organizations (NGOs) in order to shorten the time from HIV diagnosis to initiation of antiviral therapy, improve patients’ adherence, and ameliorate antiretroviral treatment (ART)-related outcomes.

**Methods:**

All newly diagnosed human immunodeficiency virus (HIV) cases at Beijing YouAn Hospital from January 2012 to December 2013 were selected as the control group, while all newly diagnosed HIV-infected patients from January 2015 to December 2016 were selected as the intervention group, receiving the comprehensive case management model.

**Results:**

4906 patients were enrolled, of which 1549 were in the control group and 3357 in the intervention group. The median time from confirming HIV infection to ART initiation in the intervention group was 35 (18–133) days, much shorter than the control group (56 (26–253) days, *P <* 0.001). Participants in the intervention group had better ART adherence compared to those in the control group (intervention: 95.3%; control: 89.2%; *p <* 0.001). During the 2 years’ follow-up, those receiving case management were at decreased odds of experiencing virological failure (OR: 0.27, 95%CI: 0.17–0.42, *P* < 0.001). Observed mortality was 0.4 deaths per 100 patient-years of follow-up for patients in the control group compared with 0.2 deaths per 100 patient-years of follow-up in the intervention group.

**Conclusions:**

People living with HIV engaged in the comprehensive case management model were more likely to initiate ART sooner and maintained better treatment compliance and improved clinical outcomes compared to those who received routine care. A comprehensive case management program could be implemented in hospitals across China in order to reduce the HIV disease burden in the country.

## Background

Human immunodeficiency virus (HIV) and acquired immune deficiency syndrome (AIDS) continue to be global health threats. According to the Joint United Nations Programme on HIV/AIDS (UNAIDS), 37.9 million people were living with HIV (PLWH), 1.7 million people were newly infected with HIV, and 770,000 people died from AIDS related illness in 2018 [1]. This is reflective of China’s epidemic as well, where new cases continue to be identified. The latest AIDS epidemic report estimated approximately 1.25 million PLWH in China by the end of 2018, with almost 80,000 new infections occurring every year [2]. the disease impacts almost more than one million people and is a serious public health and social problem [3].

The advent of antiretroviral treatment (ART) remains the most effective way to treat HIV and reduce transmission [4, 5]. In China, all people living with HIV are provided free ART therapy through the “Four Frees and One Care” program, which began in 2003. Initially, this program provided free ART mainly focus on PLWH whom had CD4 cell counts less than 200/μl in 2004, however, this changed to less than 350/μl in 2008 and less than 500/μl in 2014. Patients with higher CD4 cell counts were treated with ART when they made a special request to the providers [6]. However, currently, because of the undisputed benefits of ART, international guidelines recommend antiretroviral therapy for all patients with HIV infection regardless of CD4 cell count, and therefore our center has adaopted these guidelines [7–10]. Furthermore, the initiation of treatment as soon as possible after confirmation of HIV infection, known as “Rapid ART,” has been supported by various international groups, including the World Health Organization (WHO) [11]. Under current conditions of an absence of a vaccine or cure, “Rapid ART” can help control the HIV epidemic [12, 13].

However, the current standard-of-care pathway from initial HIV screening to starting ART in China is convoluted, involving multiple hospital visits and separate blood draws [14, 15]. In addition, most of the new diagnosed patients are reluctant to go to immunodeficiency clinics seeking ART, as they are asymptomatic. It was reported that it usually takes months before a new diagnosed patient starts treatment [16–19], and several patients lost during this period. Furthermore, with the increasing number of newly infected patients, and with the wide spread availability of free ART, and the associated benefit of prolonged survival time for the patients, more and more people are opting to receiving ART in China [12, 20]. Inspite of free ART, the HIV associated disease burden remains and continues to strain the existing healthcare system. Thus, innovative and effective HIV case management models are urgently needed in China [14, 16, 17].

Case management has been advocated as a strategy to meet the need for supportive services, to improve utilization of ambulatory services, and prevent/decrease unsatisfactory clinical outcomes for people infected with HIV [21]. The literature is scant however, on the impact of a comprehensive case management models on the time to ART initiation as it relates to ART-related outcomes in China. We report the establishment of a comprehensive case management model for HIV care that includes a coordinated efforts among doctors, nurses, and local non-governmental organizations (NGOs) and evaluated its impact on early ART and ART-related outcomes, including retention in care, virologic failure, and mortality.

## Methods

### Study population

This was an observational study of confirmed HIV-positive patients attending Beijing Youan Hospital, Capital Medical University, in Beijing, China from 2012 to 2016. Beijing Youan Hospital is one of the largest infectious disease hospitals in China, where more than half of all PLWH in Beijing receive care and ART services. HIV-positive patients attending Youan Hospital from January 2012 to December 2013 (prior to the implementation of the case management model) were selected as the control group, while all newly diagnosed HIV-infected patients from January 2015 to December 2016 (after the case management model was implemented) were selected as the intervention group.

Patients were eligible for the study if they met the following inclusion criteria: 1) received a positive HIV-1 test, 2) ≥18 years old, 3) attending Youan Hospital for HIV-related care, and 4) initiated treatment either during 2012–2013 or 2015–2016. Patient data, including demographic data, date of HIV diagnosis, date of ART initiation, and ART adherence were downloaded from the HIV/AIDS comprehensive response information management system (CRIMS), managed by Youan Hospital. Patients who had been previously treated, pregnant, not eligible for free ART, had opportunistic infections, and patients with incomplete data, such as a lack of baseline CD4 cell counts, HIV diagnosis date, or date of ART initiation, were excluded. This study was approved by the Beijing Youan Hospital Research Ethics Committee (No. 2019–057).

### Study design

All participants in the study received free ART according to the guidelines of the national “Four Frees and One Care” program. The control group received routine-HIV care from Youan hospital, including ART guidance, psychological support, health education, and laboratory follow-up. Those in the intervention group received the extended “doctor/nurse/NGO” case management model during the first 3 months of their care. This “doctor/nurse/NGO” case management model is a client-centered, strengths-based model aimed at meeting the medical and social needs of HIV-infected individuals. In this model, based on routine follow-up care, a handful of activities were conducted. First, with the help of NGOs, healthcare workers gave lectures every 2 weeks for people who had high risk of HIV infection in order to raise their awareness of the benefits of early treatment. Second, NGOs, in cooperation with medical workers, provided additional outreach on ART adherence. Members from these organizations also helped patients who had just been diagnosed with HIV finish the necessary blood tests needed before initiating ART and accompanied them to the hospital. Third, if a participant screened positive for HIV, a case manager would hold a patient assessment in a private room, providing psychological support, discussing the benefits of early ART treatment. If a participant was confirmed positive for HIV, the case manager met with the participant again in private, informing the participant of his/her status and offering support, helping them acquire more homecare. The case manager and volunteer from the NGO contacted the patients every 2 days within the first week of the intervention, and then every week for the remainder of the first month, followed by every 2 weeks for the next 2 months, by WeChat. They discuss any patient needs or discomforts and encourage them to take their medication on time.

All necessary data, including demographic characteristics (age, gender, education level, route of HIV transmission), treatment status, initial ART regimens, and follow-up status, were collected from the Beijing Youan Hospital HIV/AIDS antiviral treatment system. The ART rate, pre-treatment interval, adherence, virologic failure, retention rate, and mortality rate were compared between the intervention and control groups after 2 years of follow-up.

ART rate was calculated as the number of patients initiating ART divided by number of new patients diagnosed. Adherence to ART was a self-reported measure combined with nurses counting pills, defined as the number of times a participant reported missing a dose in the last month. Adherence to ART of every follow-up was based on the doses the patient had taken divided by expected number of in the last 30 days prior to the interview. The total adherence was measured by average all the adherence percentage of every follow-up. Participants with≥95% adherence were classified as having “good adherence” while those that took below 95% of their ART were classified as having poor adherence [22].

The pre-treatment interval was the time between confirmation of HIV and initiation of ART treatment which was defined as the number of days from when a participant tested positive for HIV using a Western Blot test to when he/she initiated treatment. Virologic failure was defined as two consecutive plasma HIV ribonucleic acid (RNA) tests of > 200 copies/ml after 24 weeks of receiving ART [23]. Based on the National Free AIDS Antiretroviral Therapy program, follow-up status for adults on ART is divided into five categories: on treatment, loss to follow-up, death, transfer to other treatment centers, and withdrawal [23]. In this study, died, loss to follow-up, and withdrawal were all defined as stopping follow-up. Retention rate was calculated as the number of patients still on treatment divided by the number of patients whom initiated ART.

All participants were followed at 2 weeks, 1 month, 2 months, 3 months, and each 3-month period until 2 years after anti-viral treatment initiation. ART adherence, routine blood work, and other tests were conducted according to Chinese clinical guidelines at each follow-up.

### Statistical analysis

Baseline and follow-up data were downloaded from CRIMS for analysis. Continuous variables were described using median and inter-quartile ranges (IQR), while categorical variables were described by percentages. The Chi-square test was used for categorical variables and the Mann-Whitney test was used for continuous variables. The cumulative probability of ART retention was calculated every 3 months after starting ART by life table analysis. A multiple logistic regression model and linear regression model were built to explore the effect of using the YouAan hospital management model on virologic failure and the pre-treatment interval, respectively. The predictors included in the multivariate model were selected based on a significance level of *P* < 0.1 in the univariate analyses or other factors that may have an impact on the failure of antiretroviral treatment. A Kaplan-Meier plot was used to assess the effect of case management on long-term survival among patients.

## Results

### Baseline characteristics of study participants

**Fig. 1:**
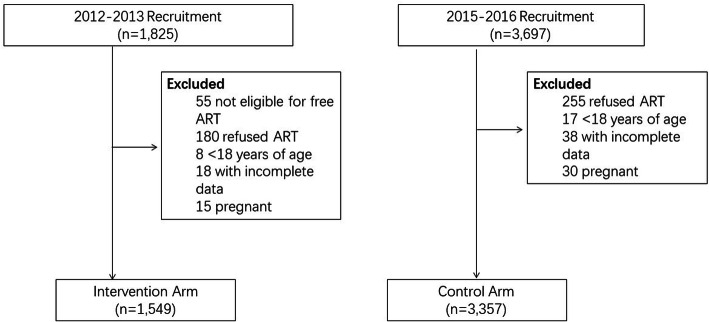
Study design

1825 patients were newly diagnosed with HIV from January 2012 through December 2013 and 3697 patients were diagnosed with HIV from January 2015to December 2016. 276 and 340 patients were excluded from the two groups, respectively, for a total of 4906 patients enrolled in the study. 1549 patients who initiated ART from January 2012 to December 2013 were selected as the control group, while 3357 patients who initiated ART from January 2015 to December 2016 were selected as the intervention group (Fig. 1). Of all participants, the median age was 35 years, the majority (96.1%), were male, 69.3% were married, and 87.4% reported a homosexual route of infection (Table 1). There were 89% patients were categorized with Stage I or II clinical infection, and approximately 62% of patients had an average CD4 cell count of under 350/μl. Age, gender, marital status, route of HIV transmission, WHO clinical stage, viral loads and baseline CD4 cell count were statistically significantly different (*p* < 0.001) between the control and intervention groups. Patients in the intervention group were younger and had higher CD4 cell counts. Additionally, more participants in the intervention group were married and reported a homosexual route of HIV transmission.

**Table 1:** Demographic characteristics of the study participants at baseline

Variable	Total (*n* = 4906)	Control group (*n* = 1549)	Intervention group (*n* = 3357)	*P*-value
**Age (Median, IQR)**	35 (30–43)	37 (32–46)	33 (29–40)	< 0.001
< 30	1181 (24.1)	165 (10.7)	1016 (30.3)	
30–49	3100 (63.2)	1136 (73.3)	1964 (58.5)	
50+	625 (12.7)	248 (16.0)	377 (11.2)	
**Gender**				< 0.001
Male	4716 (96.1)	1467 (94.7)	3249 (96.8)	
Female	190 (8.3)	82 (5.3)	108 (3.2)	
**Marital status**				< 0.001
Married	3400 (69.3)	937 (60.5)	2463 (73.4)	
Cohabitating	1279 (26.1)	525 (33.9)	754 (22.5)	
Single	203 (4.1)	77 (5.0)	126 (3.8)	
Separated/divorced	24 (0.5)	10 (0.6)	14 (0.4)	
**Route of transmission**				< 0.001
Blood transfusion	75 (1.5)	23 (1.5)	52 (1.5)	
Homosexual	4289 (87.4)	1282 (82.8)	3007 (89.6)	
Heterosexual	329 (6.7)	152 (9.8)	177 (5.3)	
Mother to child/other	213 (4.3)	92 (5.9)	121 (3.6)	
**WHO Clinical stage**^2^				< 0.001
Stage I and Stage II	4368 (89.0)	1292 (83.4)	3076 (91.6)	
Stage III and Stage IV	538 (11.0)	192 (16.6)	281 (8.4)	
**CD4 cell counts** **(Median, IQR)**	300 (191–416)	264 (163–351)	322 (207–442)	< 0.001
< 500	4229 (86.2)	1432 (92.5)	2797 (83.3)	
500+	677 (13.8)	117 (7.5)	560 (16.7)	
**Viral loads**^3^**(Mean ± SD)**	4.2 ± 0.83	4.2 ± 0.73	4.2 ± 0.86	0.030
**ART Regimen**				0.530
EFV/LPV/r + 3TC + AZT/TDF	4777 (97.4)	1505 (97.2)	3066 (97.5)	
Other	129 (2.6)	44 (2.8)	85 (2.5)	

*IQR* interquartile range; *WHO* World Health Organization, *ART* anti-retroviral therapy; *EFV* Efavirenz; *3TC* Lamivudine; *AZT* Zidovudine; *TDF* Tenofovir; *LPV/r* Kaletra

2 WHO Clinical Stage: a way to categorize HIV disease severity based on new or recurrent clinical events. There are 4 WHO clinical stages which range from mild symptoms (WHO clinical stage 1) to severe symptoms (WHO clinical stage 4)

3915 did not have baseline viral loads

### Impact of case management on ART-related outcomes

The ART rate was 89.8% (1590/1770) and 93.1% (3442/3697) in control and intervention group, respectively (*P* < 0.001). The median pre-treatment interval in the intervention group was 35 (18–133) days, statistically significantly shorter than in the control group (*P* < 0.001), which was 56 (26–253) days (Table 2). More participants in the intervention group had good ART adherence compared to those in the control group (intervention: 95.3%; control: 89.2%; *P* < 0.001), while more control participants experienced virological failure (intervention: 0.9%; control: 3.9%, *p* < 0.001). Observed mortality was 0.7 deaths of follow-up for patients in the control group compared with 0.3 deaths in the intervention group (*P* < 0.001). Similar results were obtained among patients with CD4 cells less than350/μl (Table 2).

**Table 2:** Antiviral therapy related outcomes of the participants, by study arm

Indicator	All Participants			Participants with CD4 Less Than 350/μL		
	Control group (*n* = 1549)	Intervention group (*n* = 3357)	*P*-value	Control group (*n* = 1158)	Intervention group (*n* = 1983)	*P*-value
Virological failure	60 (3.9)	31 (0.9)	< 0.001	51 (4.4)	27 (1.4)	< 0.001
Retention in care	1466 (94.6)	3159 (94.1)	0.449	1096 (94.6)	1774 (93.7)	0.290
pre-treatment interval (days)^1^	56 (26–253)	35 (18–133)	< 0.001	48 (25–252)	35 (17–106)	< 0.001
Good ART adherence	1381 (89.2)	3199 (95.3)	< 0.001	949 (82.0)	1817 (96.0)	< 0.001
Death (/per 100 year)	11 (0.4)	10 (0.2)	< 0.001	11 (0.9)	10 (0.5)	< 0.001

1pre-treatment interval, days from when a participant tested positive for HIV using a Western Blot test to when he/she initiated treatment

### Factors associated with virologic failure

The results of the multivariate logistic regression are summarized in Table 3. Participants who received case management were at decreased odds of virological failure (OR: 0.27, 95% CI: 0.17–0.42, *p* < 0.001). Additionally, participants with more severe disease, specifically WHO stage III or stage IV were at increased odds of virological failure (OR: 1.94, 95% CI: 1.17–3.21, *p* < 0.01 (Table 3).

**Table 3:** Factors associated with virological failure among all participants

		Crude model		Adjusted model	
Variable	Events/N (%) or Median (IQR)	OR (95% *CI)*	*P*-value	OR (95% *CI)*	*P*-value
**Case management**					
No case management	60/1549 (3.9)	Reference			
With case management	31/3357 (0.9)	0.23 (0.15–0.36)	< 0.001	0.27 (0.17–0.42)	< 0.001
**WHO Clinical stage**					
Stage I and stage II	69/4368 (1.6)	Reference		Reference	
Stage III and stage IV	22 /538 (4.1)	2.67 (1.63–4.33)	< 0.001	1.94 (1.17–3.21)	0.010
**CD4 cell counts**					
< 500	83/4229 (2.0)	Reference		Reference	
500+	8/677 (1.2)	0.60 (0.29–1.24)	0.167	0.89 (0.42–1.87)	0.756

*OR* Odds Ratio; *CI* Confidence Interval; *WHO* World Health Organization

### Factors associated with the pre-treatment interval

As shown in Table 4, the days from HIV diagnosis to ART initiation was only associated with the implementation of the comprehensive case-management, and it was negatively correlated with the days between HIV diagnosis and ART initiation.

**Table 4:** Factors associated with the pre-treatment interval among all participants

Variable	β (95% *CI)*	*P-*value	β (95% *CI)*	*P-*value
Case management	−30.62 (− 59.04, -2.20)	0.035	− 30.36 (− 58.78,-1.95)	0.036
ART Regimen	83.26 (0.69,165.82)	0.048	82.47 (− 0.07,165.01)	0.050

*ART* antiretroviral treatment; *CI* confidence interval

### Mortality

**Fig. 2:**
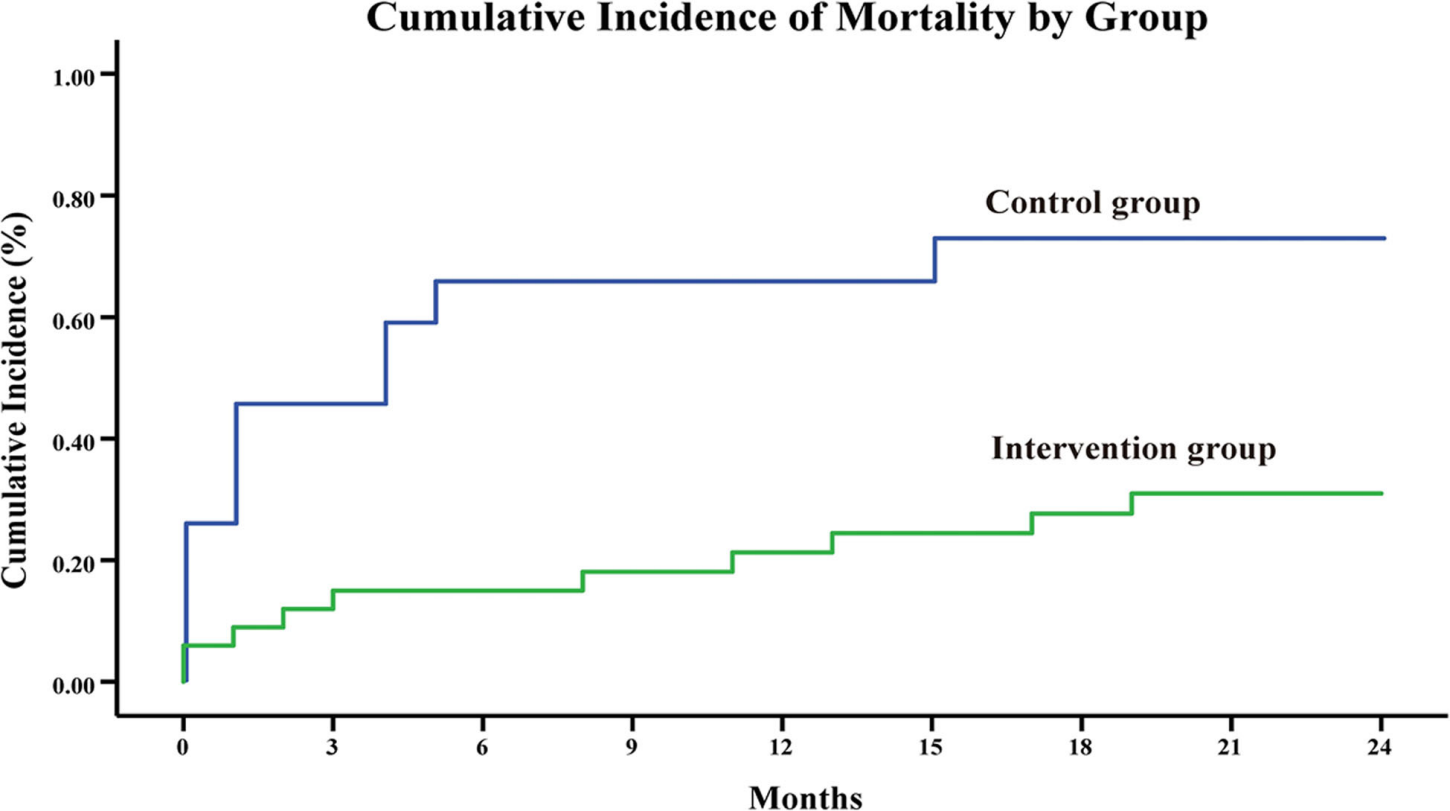
Kaplan-Meier survival plot among patients with and without case management. This figure shows the cumulative incidence of mortality in the control group (blue line) and the intervention group (green line). The cumulative incidence of mortality in the control group is above the intervention group at each follow-up, indicating that the intervention group had a better survival rate throughout the study period (*P* = 0.023)

A total of 21 (0.4%) patients died during the 2 years’ study period (Fig. 2). There were 0.7 deaths for patients who did not receive case management (control group) compared with 0.3 deaths for those who received case management (intervention group). The Kaplan-Meier survival analysis summarizes the survival curve between the two groups, indicating that the control group had a higher cumulative incidence of mortality compared to the intervention group (*P* = 0.023).

## Discussion

Case management uses a client-centered, multi-faceted approach to ensure health and psychological support for people living with HIV. While research has shown the benefits of implementing case management on HIV-related outcomes, little has been done in China to assess the impact of comprehensive interventions on early ART and patient-related outcomes together. In this study, we evaluated the impact of one such strategy at Beijing Youan Hospital, one of China’s largest clinics treating more than 9000 HIV infected individuals. In this study, we found that after implementation of a comprehensive case-management program, ART-related outcomes including retention and survival increased, while the time from initial HIV diagnosis to initiation of treatment was shortened. This study provides evidence to support a case-management modality as an integral aspect to HIV care in China and could be incorporated across the country to improve patient-related outcomes and decrease the burden of disease.

In this study, the average number of days from confirmed HIV diagnosis to initiation of ART treatment was 35 days in the intervention group (those receiving case-management) and 56 days in the control group (those who did not receive case management). Early initiation of ART and linkage to care are necessary to achieving viral suppression, subsequently reducing the transmission of HIV and preventing new infections [19]. In China, the routine HIV screening process is convoluted, often involving multiple counseling sessions and blood draws, and due to lack of courage or lack of cognition or fear of side effects of the drugs [24], new diagnosed people are usually reluctant to come back to the clinic seeking treatment proactively. A study among older PLWH in Chongqing City, for example, found that the average time from HIV diagnosis to ART initiation was 6.3 months among those aged over 50, and almost 13 months for those between the ages of 18–49 [21]. A similar study from Yuxi, China determined an average of 447 days between HIV confirmation and treatment initiation [22]. During this pre-ART period, substantial mortality and loss to follow-up have been observed, especially among people with advanced HIV disease [11, 24, 25]. Recent randomized trials have indicated that shortening the time for patients to enter treatment can reduce this loss between diagnosis and treatment [26, 27], but many experts worry that shortening this “preparation time” can lead to more loss due to inadequate consultations [28]. In our study, NGO united medical staff, through establishment of communication at a personal and confidential level, HIV education and information transfer, etc. facilitates the establishment of the comprehensive case management model that resulted in fewer days between HIV diagnosis and ART initiation accompanied with better ART compliance and treatment outcome even among lower CD4 count patients. Similar findings have been reported in high-income countries, such as the United States (US). Since the AIDS epidemic began in the 1980s, the US has used case management to improve the quality of medical care, meet the needs of AIDS patients, and decrease the associated cost of care [13, 29, 30]. This has subsequently led to less unmet demand and higher rates of ART initiation in the country [31]. Thus, evidence suggests that comprehensive case management can be effective in reducing loss to follow-up and improving linkage to care, critical aspects to timely ART initiation.

Consequently, this has also led to improvements in clinical results. Specifically, in this study, the results of the multivariate logistic regression analysis indicated that patients who received the new case management model were at decreased odds of developing virological failure (*P* < 0.001), which is consistent with other findings [32–34]. More so, those receiving case management experienced consistently higher survival rates compared to those in the control group. This quicker linkage to care may have resulted in the decreased mortality in the intervention group. Mortality is also related to patient adherence, as improved ART adherence can lead to long-term viral suppression, thereby reducing morbidity and mortality [30, 34, 35]. As seen in this study and others, case management is an effective means to improving ART adherence and retention, subsequently reducing mortality. In light of this evidence, efforts to identify individuals at the beginning of their infection and streamline the process from HIV screening to ART care, such as used in this case management system, could be a beneficial tactic to include in national care guidelines. A national roll out of this multi-dimensional case management strategy could improve the care of PLWH in China and reduce the overall disease burden in China.

Some limitations to this study exist. As this was a retrospective study, confounding characteristics may be present. Even more, there is no way for us to conduct a periodical control, only a historical control can be set up. there were significant differences in baseline characteristics between the control and intervention groups, including age, gender, and route of transmission. But the historical control information we included was from the CRIMS information system, which was not upgraded during the entire study period. So the information of both groups can be obtained the same way, avoided the bias caused by the review of supplementary information. At the same time, multivariate analysis was used, in order to control for the influence of other imbalance factors on study results. Moving forward, a matched, case-control study could be implemented to further assess the impact of case management on ART initiation, adherence, and retention. Additionally, participants were restricted to those attending Beijing Youan Hospital and may not be representative of the general population living with HIV throughout China. Youan Hospital however, is one of the leading HIV care institutes in the country, where more than 9000 individuals receive care. Thus, a diverse sampling of the HIV infected population would likely be captured. Further evaluation of the case management modality in different medical departments across the country could help further supplement the findings from this study.

## Conclusion

A doctor/nurse/NGO HIV case management program is feasible and could be an effective complementary strategy to improving HIV treatment outcomes in China. In order to improve patient outcomes, further understanding of the causes for loss to follow-up should be explored. Supplementing the existing case management model with real-time feedback from patients can only further strengthen this innovative intervention and improve HIV/AIDS care throughout China.

## Data Availability

The dataset used and/or analyzed during the current study is available from the corresponding author on reasonable request.

## Abbreviations

NGOs: Non-governmental organizations
ART: Antiretroviral treatment
HIV: Human immunodeficiency virus
OR: Odds ratio
CI: Confidence intervals
AIDS: Acquired immune deficiency syndrome
UNAIDS: Joint United Nations Program on HIV/AIDS
PLWH: People were living with HIV
WHO: World Health Organization
China CDC: Chinese Center for Disease Control and Prevention
CRIMS: HIV/AIDS comprehensive response information management system
RNA: Ribonucleic acid
IQR: Inter-quartile ranges
US: United States
